# The impact of sports participation on reappraisal use during daily challenges among young adults

**DOI:** 10.3389/fpsyg.2025.1701733

**Published:** 2025-12-15

**Authors:** Wing Sze Lam, Leah H. Somerville

**Affiliations:** 1 The Division of Continuing Education, Harvard Extension School, Cambridge, MA, United States; 2 Northwest Laboratory, Department of Psychology, Harvard University, Cambridge, MA, United States

**Keywords:** competition, depression, early adulthood, emotion regulation, reappraisal, sports participation, young adults

## Abstract

**Background:**

In this retrospective study, we aimed to investigate the relationship between reappraisal use, an efficacious emotion regulation strategy, and sports participation among young adults. The primary objective was to determine whether sports participation in competitive environments is associated with reappraisal when young adults face adversity.

**Methods:**

Young adults between the ages of 18 and 25 who lived in the United States were recruited through free online research portals and paid panel services. Upon confirming their consent statements, all participants were directed to the Harvard-hosted online Qualtrics platform to self-complete the questionnaire, which covered demographics, history of sports participation, stressful life events, use of emotion regulation strategies in daily life, and the likelihood of feeling depressed.

**Results:**

This study analyzed data from 431 respondents who provided usable data. We found that young adults who engaged in sports were significantly more likely to use reappraisal in the past 3 months and in daily life compared to those who did not participate in sports. In addition, individuals who participated in competitive sports showed a greater tendency to employ reappraisal compared to those who participated in non-competitive sports. This effect held when accounting for depression symptoms, which were higher in the competitive group compared to the non-competitive group.

**Conclusion:**

Participation in sports, particularly competitive sports, is associated with more frequent use of reappraisal to manage emotions among young adults. Future research should examine the relationship between these variables to ascertain whether participation in sports influences the use of reappraisal techniques or if the ability to employ reappraisal skills impacts the decision to engage in competitive sports. Furthermore, it is essential to explore whether other factors may mediate the relationship between these two variables.

## 1 Introduction

Managing emotions can be challenging, especially for young adults transitioning into early adulthood, who often face substantial social and emotional challenges. With the increase in independence, lifestyle adjustments can constantly frustrate young adults, which increases their daily stress and emotions, including sadness, depression and anxiety (American Psychological Association [APA], 2022; Wiium et al., 2015). Coping with these emotions is crucial, and tools like emotion regulation may help individuals manage their feelings through both highs and lows.

Some common adaptive strategies include engaging in activities to divert attention away from negative thoughts (distraction; Gross, 1999), Sharing emotions with friends and family, interacting with pets to foster social connections (social support; Zhang et al., 2022), reinterpreting an adverse situation in a positive light (reappraisal; Gross, 1998), acknowledging and accepting the situation with an open attitude, and not attempting to change it (acceptance; Williams and Lynn, 2010). Conversely, many others tend to employ maladaptive strategies to internalize their negative feelings by hiding their emotions with neutral facial expressions (expressive suppression; Cutuli, 2014), blaming themselves for their mistakes and repeatedly dwelling on negative thoughts related to the situation (rumination; Nolen-Hoeksema et al., 1997), and using drugs or self-harm to avoid thinking of emotional pains (avoidance; Hayes et al., 1999). The choice and effectiveness of employing these strategies vary widely among young individuals.

Differences in preferred emotion regulation strategies can impact the effectiveness of young adults in managing stress and maintaining their psychological health. Nevertheless, the ability of young adults to utilize emotion regulation strategies is influenced by various factors, including their demographics, life experiences, and daily stressors (Wessa et al., 2024; Xu et al., 2020). These elements play a crucial role in shaping the specific strategies they choose to manage their emotions. For instance, males tend to rely on expressive suppression more than females, and the tendency to endorse reappraisal increases with age (John and Gross, 2004). On the other hand, young adults often seek social support from those with whom they have a stronger relationship in times of distress or need. They tend to confide in their families and trusted peers (Australian Institute of Family Studies, 2018).

A solid social network can provide encouragement and guidance, which enhances emotional management and resilience while also increasing self-worth and reducing anxiety and depressive symptoms (Narr et al., 2019). However, changes in social support networks (Metts and Craske, 2023) may further complicate the selection and effective implementation of these strategies. For example, when young adults encounter challenging situations in new environments, such as beginning a new job or navigating relationships, they may feel uncertain about whom to trust, making them reluctant to share personal issues with unfamiliar individuals. As such, young adults tend to employ distraction, rumination, and suppression as means of immediate emotional relief during periods of stress or adversity (Brans et al., 2013; Cooper et al., 2016), rather than reappraisal despite its association with improved mood (Brans et al., 2013; Goubet and Chrysikou, 2019) and fewer symptoms of depression (Aldao et al., 2010; Roos and Bennett, 2023). Physical activity, for instance, is an effective way to alleviate anxiety by distracting attention from distressing thoughts (Bahrke and Morgan, 1978; Mikkelsen et al., 2017).

Physical activity comes in various forms. Participating in sports is a subset of physical activity that is organized and repetitive with a specific goal to achieve (Caspersen et al., 1985). While sports participation offers diverse means to manage emotional responses, research on sports activities has focused more on the positive impact of emotion regulation on sports performance in the past decades (Balk et al., 2013; Robazza et al., 2004). Recently, researchers have gradually shifted their focus to paying more attention to the association of sports participation and enhanced coping skills when facing life’s challenges and stressors (Appelqvist-Schmidlechner et al., 2020). Some studies focus on reducing the risks of depression and anxiety across various age groups, including adolescents and adults (Mahindru et al., 2023; Schuch et al., 2018), as well as enhancing self-efficacy and self-esteem (Joseph et al., 2014; Sani et al., 2016). Other research has shown that sports participation can offer emotional benefits by enhancing emotional regulation skills (Liu et al., 2022). However, a notable gap remains in research regarding the determinants of sports participation that influence the tendency to use emotion regulation strategies, particularly reappraisal. It is also crucial to investigate whether any external factors of sports participation influence the tendency to use reappraisal.

First, the concept of reappraisal and sports participation shares a commonality: repetition. As noted by Caspersen et al. (1985), participating in sports involves consistent practice and memorization. Prolonged participation in sports, particularly in competition, strengthens resilience by encouraging individuals to identify and reinterpret positives in failures, ultimately enhancing both performance (Secades et al., 2016) and mental well-being (Hosseini and Besharat, 2010). Given that the competitive nature of sports can promote mood control and enjoyment by boosting motivation and a sense of accomplishment (Tamminen et al., 2016), this learning process of coping with failure may be crucial for adapting to real-life challenges. Similarly, the skill of reappraisal can be refined through practice, allowing individuals to regulate their emotions more effectively. The study by Denny and Ochsner (2014) highlighted that reappraisal was effective in reducing participants’ negative emotions over time through practice. As such, it is essential to explore if the time duration and competitiveness of sports influence the association between reappraisal and sports participation, given that practice is vital in both areas.

Second, with the overwhelming daily stressors, young adults can quickly become exhausted and frustrated. There is a concerning trend of increasing emotional distress among young adults, such as stress, anxiety, and sadness (Gallup, 2023). As individuals age and face more adverse life events (Mann et al., 2014), young adults often experience heightened pressure, increasing the likelihood of developing depression, which hampers effective emotional management. Conversely, sports participation can enhance resilience when encountering challenges (Hosseini and Besharat, 2010). Reappraising stressful events from different perspectives can also mitigate the negative emotional impact of their initial reaction (Denny and Ochsner, 2014; Lennarz et al., 2019). This raises important questions about whether stress influences sports participation and reappraisal use, as well as their relationship. Investigating their connection could provide valuable insights into whether sports participation facilitates the use of reappraisal, particularly under stress.

Third, numerous studies have shown no gender differences in the habitual use of cognitive reappraisal (Gross and John, 2003; Perchtold et al., 2019). Nevertheless, more females than males reported feelings of sadness and worry, and the gap has increased over the past decade (Centers for Disease Control and Prevention [CDC], 2023). Similarly, Johansen et al. (2021) have reaffirmed in their research that females exhibit higher levels of mental distress than males. Females appear to reach the highest level of distress at 30% in their early twenties, compared to the peak at approximately 15% for males at the age of 30. Additionally, females are more likely to drop out of sports, and this trend increases as they age (Canadian Women and Sport, 2020) due to lower motivation levels (Portela-Pino et al., 2019). This uncertainty has prompted researchers to investigate whether other emotion regulation strategies are at play. Goubet and Chrysikou (2019) found that college-aged women in the United States tended to use a range of emotion regulation strategies more frequently than men in the same age group. For instance, females were more likely to engage in self-blame and seek social support in romantic situations. They also utilized more social support and problem-solving strategies in academics than their male counterparts.

The present study aimed to investigate the impact of sports participation on the tendency to use reappraisal in young adults when facing daily challenges. We were particularly interested in whether the competitive nature of sports plays a role in this tendency and its relationship with the use of reappraisal. We asked *N* = 501 young adults aged 18–25 in the United States to complete measures of their sports participation over the past 12 months, stressful events experienced in the past 3 months, clinical symptoms of depression in the past 2 weeks, and their use of emotion regulation strategies, particularly reappraisal. Additionally, as young adults participate in sports in various ways, they were asked to specify sport types they engage in, such as school or club team (team sports), social playing with friends that are focused on socializing rather than training or coaching in competitive or non-competitive settings (social sports) and playing individual sports that one participates in by oneself (solo sports).

The study proposed four hypotheses. The first hypothesis posited that sports participation, relative to non-participation, would be associated with reappraisal use. The second hypothesis suggested that young adults playing sports in competitive settings would use reappraisal skills more frequently than those in non-competitive sports or non-participants. The third hypothesis proposed that the relationship between sports participation and reappraisal use would be stronger with a longer duration of sports participation. The fourth hypothesis was that females would participate in sports less frequently and thus use reappraisal less often.

## 2 Materials and methods

### 2.1 Participants

A total of 501 volunteers completed surveys from young adults aged 18–25 residing in the United States. After filtering out potential bots and incomplete and unrealistic responses (e.g., recorded identical scores across all scale questions) to ensure reliable data, *n* = 431 samples were used in the final analysis. Participants were recruited between February and March 2024 through free online research portals, The Study Pool of the Department of Psychology at Harvard University, and Kantar’s LifePoints survey panels paid service. A power analysis was conducted to determine the sample size before the study began. For a one-way between-subjects ANOVA (five groups, alpha = 0.05), G*Power 3.1.9 suggested the minimum sample size to yield a statistical power of at least 0.8 and a medium effect size of 0.25 was 270 for computing analysis to test the second hypothesis in relation to sports competitive and non-competitive settings (Faul et al., 2009). This minimum sample size was used as a benchmark during data collection.

The survey incentive for Kantar’s LifePoints panelists was compensated for completing the study based on the LifePoints panel’s rewards program terms. Harvard students recruited from the Study Pool of the Department of Psychology were awarded 0.5 SONA credits for every half hour of their involvement. Furthermore, other participants who provided valid email addresses could win a $10 e-gift card through a raffle. All research procedures were approved by Harvard University’s Committee on the Use of Human Subjects, consistent. Participants provided informed consent prior to participating.

### 2.2 Design

This study employed a survey-based design to examine the use of emotion regulation strategies among young adults when facing adversity in their daily lives. We also focused on the least-used strategy, reappraisal, and investigated its relationship with sports participation. Additionally, the data of (1) demographic information; (2) sports participation, including frequency and duration of participation in the past year; (3) prevalence of negative life events that occurred in the past 3 months; (4) emotion regulation strategies used, as well as their tendency to regulate emotions by cognitive reappraisal and expressive suppression; and (5) endorsement of depression symptoms over the past 2 weeks were collected.

### 2.3 Instruments

After providing the informed written consent, survey participants self-completed the online questionnaires covering five sections.

#### 2.3.1 Sociodemographic

##### 2.3.1.1 Demographic questionnaire

This questionnaire has nine questions about age, gender, education, marital status, race and ethnicity, and employment status. Its purpose is to collect the essential characteristics of the sample to understand the group’s composition.

#### 2.3.2 History of sports participation

##### 2.3.2.1 Sports participation questionnaire

A closed-ended questionnaire with nine multiple-choice questions was developed to gather information on sports participation, which included specifying sports type (i.e., school or club team sports, competitive and non-competitive social sports and solo sports) and participation patterns such as how frequently (ranging from daily to once-in-a-while) and for how long (ranging from less than a month to over 3 years) individuals had participated in each sport type in the past 12 months. This scale was custom-developed to gather data on sports involvement, aiming to determine participants’ physical activity levels. Sports participation in this study included both exercise (planned activities for fitness) and sports (competitive or social physical activities that follow specific rules). The term “sports” was used throughout the rest of this paper.

Based on responses to this questionnaire, participants were assigned one of five sports participation categories: (1) team sports, (2) Social sports in competitive settings, (3) Social sports in non-competitive settings, and (4) Solo sports. In this study, physically active young adults were defined as those who played sports at least once a week for at least 30 min each time. Individuals who did not meet these criteria were defined as inactive and were included in the (5) sports non-participant group.

#### 2.3.3 Stressful challenges

##### 2.3.3.1 Negative life events scale for students (NLESS)

The NLESS (Buri, 2018) evaluated the presence of stressful life events relevant to the college-aged population. Participants responded with a code 1 for “yes” and 0 for “no” if they had experienced any of the listed stressful life events during the preceding 3 months. The items related to abuse, violence, sexual topics, and legal matters (i.e., attributes 7, 8, 9, 20, 22, and 24 in the original version) were removed from this study to minimize risk to the participants. The total number of items was reduced to 19. The sum of all items reflects a score for negative life events, with higher scores indicating a greater number of negative life events experienced in the past 3 months.

#### 2.3.4 Emotion regulation

##### 2.3.4.1 Emotion regulation strategies (ERS)

The present study used the Emotion Regulation Strategies questionnaire (Brans et al., 2013) to measure the use of six emotion regulation strategies in daily life. Participants were asked to self-report on the extent of their engagement in each of six emotion regulation strategies on a 6-point Likert scale, ranging from 0 (not at all) to 5 (very much so). Each strategy was measured using a single item, including “I have engaged in activities to distract myself from my feelings” (distraction), “I have avoided expressing my emotions” (expressive suppression), “I have changed the way I think about what causes my feelings” (reappraisal), “I have calmly reflected on my feelings” (reflection), “I couldn’t stop thinking about my feelings” (rumination), and “I have talked about my feelings with others” (social sharing). Each score was calculated independently. A higher score indicates greater use of a specific emotion regulation strategy, whereas lower scores indicate less frequent use.

##### 2.3.4.2 Emotion regulation questionnaire (ERQ)

The ERQ (Gross and John, 2003) consisted of 10 items about the tendency to use reappraisal and suppression strategies. The cognitive reappraisal subscale contained six statements (e.g., “When I want to feel less negative emotions, I change the way I think about the situation”) and the expressive suppression subscale contained four statements (e.g., “I keep my emotions to myself.”). Responses were recorded on a 7-point Likert scale ranging from 1 (strongly disagree) to 7 (strongly agree). The scoring methodology involves computing the means of all the scores within each subscale of cognitive reappraisal and expressive suppression. A higher mean score indicates a more pronounced reliance on the respective emotion regulation strategy.

We employed two measures to compare results and confirm the consistency of the findings if both measures yielded similar results.

#### 2.3.5 Endorsement of depression symptoms

##### 2.3.5.1 Depression screen questionnaire (PHQ)

The PHQ (aka Patient Health Questionnaire; Centers for Disease Control Prevention [CDC] and National Center for Health Statistics [NCHS], 2021) was an excerpt from the National Health and Nutrition Examination Survey (NHANES) conducted by the National Center for Health Statistics of the Centers for Disease Control and Prevention to examine adults’ and children’s health and nutritional status in the United States. The original nine-item PHQ instrument is used to screen for depression. Each item is rated on a scale of 1 (not at all) to 4 (nearly every day). In this study, the option of “Don’t know” and the question related to self-harm and suicidal ideation were removed from the questionnaire. Participants were asked to rate the frequency of their depressive symptoms over the past 2 weeks (Centers for Disease Control Prevention [CDC] and National Center for Health Statistics [NCHS], 2021; Kroenke et al., 2001). The scores were summed in each subscale, with a higher score indicating a greater likelihood of experiencing depression symptoms (DS) over the past 2 weeks.

### 2.4 Procedure

An initial pre-screening required the participants to confirm that they were 18–25 years old and resided in the United States through two binary screening questions. Ineligible participants were thanked and redirected away from the survey.

Eligible participants reviewed informed consent statements outlining the survey’s purpose, potential risks, time requirements, and their rights. After agreeing to participate, they were directed to complete the study on the Harvard-hosted Qualtrics online platform. The study comprised five sections and took approximately 30 min to complete. Participants could opt out at any time, and all information provided was kept confidential.

### 2.5 Statistical analysis

After removing any missing data, potential bots and frauds, the data were analyzed using JASP 0.18.3 and IBM SPSS Statistics 29. The effect sizes were calculated using Cohen’s d and Pearson’s r to gauge the strength of the relationship between the dependent and independent variables in all statistical tests.

First, to explore whether sports participation in relation to non-participation was associated with the use of reappraisal, the study conducted independent sample *t*-tests to compare the scores of cognitive reappraisal and expressive suppression in ERQ between sports participants and non-participant groups. Also, as all dependent variables in ERS failed the normality test, a Mann-Whitney U-test was conducted to compare the scores of each emotion regulation strategy (reappraisal, reflection, rumination, social sharing, expressive suppression, and distraction). A significant main effect of sports participation on reappraisal use in both tests would provide evidence supporting the first hypothesis: (1) Sports participation is associated with the tendency to use reappraisal. Individuals who participate in sports are more likely to use reappraisal than non-participants.

Second, to investigate the role of competitiveness in sports, we conducted one-way ANOVA tests comparing three groups: competitive, non-competitive, and non-participants. After ANOVA revealed a significant overall effect, we used a *post-hoc* test, Tukey’s Honest-Significant-Difference (Tukey’s HSD), to compare mean differences across three levels of sports participation regarding reappraisal use and identify where those differences occurred. A significant result from Tukey’s HSD analysis would provide support for the second hypothesis: (2) Young adults in the competitive group would use reappraisal skills more frequently than those in non-competitive and non-participant groups.

Third, a set of independent *t*-tests and chi-square tests were performed to investigate potential gender differences in depression symptoms and duration of playing sports across competitive, non-competitive, and non-participation groups. Finally, multiple linear regressions with the forced entry method were used to investigate whether depression symptoms, number of negative life events and gender were significant predictors of reappraisal use. Reappraisal was treated as a continuous dependent variable, as measured by the Emotion Regulation Questionnaire (ERQ), which computes a mean score across six Likert-scale items. The analysis aimed to evaluate the overall model fit and the relative contribution of each predictor to the variance in reappraisal scores. Depression symptoms, negative life events and gender were considered potential covariates and entered into the first block for statistical control. This was followed by an analysis of covariance (ANCOVA) to test the main and interaction effects of sports participation on reappraisal use while controlling for the effects of covariates if a significant percentage of variance was explained. The significant effect of these tests would provide support for the third and fourth hypotheses: (3) The more years of participation in competitive sports, the stronger the relationship between sports participation and reappraisal use, and (4) Females would engage less in sports activities and use reappraisal less frequently compared to males.

## 3 Results

### 3.1 Sample characteristics

Of the 629 responses, 128 were incomplete, and 70 were unreliable data or potential bots with a Q_RecaptchaScore of 0.5 or less. The final sample size for analysis was 431, including 228 females (53%), 195 males (45%), and eight others indicated as transgender males or females or preferred not to disclose. The age range was from 18 to 25, with a mean of 22.21 and a standard deviation of 2.08. More than half (51.3%) identified as White, and 76.8% as single. The majority (75.9%) had completed high school or higher education. The sample also comprised 36.4% full-time students and 37.4% full-time employed. A chi-square test was conducted to analyze the distribution of the sample characteristics among sports participants and non-participants. Whereas there were no statistically significant differences in gender (*p* = 0.236), marital status (*p* = 0.458), and race (*p* = 0.062), education levels and occupation status differed statistically significantly between the two groups. For instance, sports participants were more likely to obtain higher education than non-participants, χ^2^ (6, *N* = 431) = 29.95, *p* < 0.001, Cramer’s *V* = 0.26. Moreover, sports participants were more likely to be currently studying, χ^2^ (2, *N* = 431) = 38.41, *p* < 0.001, Cramer’s *V* = 0.30, and working, χ^2^ (2, *N* = 431) = 14.83, *p* < 0.001, Cramer’s *V* = 0.19. The details of the socio-demographic profile is shown in Table 1.

**TABLE 1:** Total sample characteristics, *n* (%).

Demographic variables	Total (*N* = 431)	Sports-NP (*n* = 142)	Sports-P (*n* = 289)
Age, years: *M* (SD)	22.21 (2.08)	22.32 (2.07)	22.17 (2.09)
**Gender**			
Female	228 (53%)	83 (58%)	145 (50%)
Male	195 (45%)	56 (39%)	139 (48%)
Others	8 (2%)	3 (2%)	5 (2%)
**Marital status**			
Single, never married	331 (77%)	106 (75%)	225 (78%)
Married	57 (13%)	23 (16%)	34 (12%)
Separated	3 (1%)	0 (0%)	3 (1%)
Others	40 (9%)	13 (9%)	27 (9%)
**Ethnicity/race**			
American Indian/ Alaska Native	6 (1%)	3 (2%)	3 (1%)
Asian	32 (7%)	6 (4%)	26 (9%)
Black/ African American	115 (27%)	34 (24%)	81 (28%)
Hispanic/ Latino	113 (26%)	37 (26%)	76 (26%)
Native Hawaiian	2 (0.5%)	0 (0%)	2 (1%)
White	221 (51%)	79 (56%)	142 (49%)
Mixed	26 (6%)	5 (4%)	21 (7%)
Others	26 (6%)	14 (10%)	12 (4%)
**Education level**			
Postgraduate	12 (3%)	1 (1%)	11 (4%)
College graduate	72 (17%)	19 (13%)	53 (18%)
Some college	165 (38%)	37 (26%)	128 (44%)
High school graduate	162 (38%)	76 (54%)	86 (30%)
Some high school	13 (3%)	6 (4%)	7 (2%)
Preschool to middle school	3 (1%)	2 (1%)	1 (0.3%)
No schooling	4 (1%)	1 (1%)	3 (1%)
**Occupation**			
FT student	157 (36%)	32 (23%)	125 (43%)
PT student	80 (19%)	16 (11%)	64 (22%)
FT employed	161 (37%)	50 (35%)	111 (38%)
PT employed	134 (31%)	31 (22%)	103 (36%)
Neither student nor employed	75 (17%)	48 (34%)	27 (9%)

All values in the table are presented as frequency (percent), except for age, which is presented as mean (standard deviation). Sports-P and Sports-NP represent sports participants and non-participants, respectively.

According to self-reported data from 67% of individuals (*n* = 289) who participated in sports in the past 12 months, most of their time was spent in the following activities: 14.4% played for school teams, while 10% played for club teams, 11.8% engaged in competitive social sports with friends, 13.7% participated in non-competitive social sports with friends, and 17.2% played sports alone. Approximately 56.4% of the participants (*n* = 163) engaged in sports for 12 months or less, whereas 43.6% (*n* = 128) were active for over a year.

Young adults reported experiencing a range of negative life events (NLE) in the past 3 months, from zero to twelve, with a mean of 2.42 and a standard deviation of 2.28. Since the normality assumption of the NLE was not met, a Mann-Whitney U-test was conducted. The results of the descriptive statistics show that the sports participants group had a higher mean for experiencing NLE (*M* = 2.47) than the non-participants group (*M* = 2.32). However, the difference between both groups with respect to the experienced NLE in the past 3 months was not statistically significant, *z* = −0.56, *p* = 0.578, η^2^ < 0.001.

Furthermore, to better understand the likelihood of experiencing depression symptoms (DS) within the sample, the scoring involved summing all the scores in each subscale of the Depression Screen Questionnaire (PHQ). A higher score indicates a more significant experience of DS over the past 2 weeks. According to the data, the average DS score reported by young adults in this sample was 12.3 (SD = 6.05). The equality of variances in Levene’s Test was confirmed (*F* = 1.50, *p* = 0.222). The results of the Independent Samples *t*-test indicated no significant difference in experiencing DS between sports participants (*M* = 12.17) and non-participants (*M* = 12.57), *t*(406) = 0.62, *p* = 0.536, *d* = 0.07, 95% CI [−0.86, 1.65]. This implies that the young adults in this study encountered similar daily challenges and stressors despite participating in sports.

### 3.2 The Relationship between sports participation and reappraisal

Two specific measures were conducted to evaluate the tendency to use reappraisal as a strategy for regulating emotions. The first measure was the “Use of Six Emotion Regulation Strategies in Daily Life” (ERS; Brans et al., 2013), which required participants to indicate the tendency with which they employed distraction, expressive suppression, reappraisal, reflection, rumination, and social sharing in their daily lives. The second measure was the Emotion Regulation Questionnaire (ERQ; Gross and John, 2003), which assessed how individuals manage their emotions daily through cognitive reappraisal and expressive suppression.

Before delving into the comparison of sports participation groups in relation to reappraisal, the preliminary ERS data screening showed that scores in both groups met the assumption of homogeneity of variance with Levene’s test in reflection (*F* = 0.15, *p* = 0.699), reappraisal (*F* = 0.58, *p* = 0.499), rumination (*F* = 0.92, *p* = 0.337), social sharing (*F* = 0.995, *p* > 0.319), expressive suppression (*F* = 0.62, *p* = 0.43), except distraction (*F* = 7.66, *p* = 0.006). In addition, the normality test failed for all dependent variables in ERS. Therefore, a Mann-Whitney U-test was performed to evaluate the difference in each emotion regulation strategy. The reflection (*z* = −3.36, *p* < 0.001, η^2^ = 0.03), reappraisal (*z* = −3.03, *p* = 0.002, η^2^ = 0.02), and distraction (*z* = −2.60, *p* = 0.009, η^2^ = 0.02) in the sports participants group were statistically significantly higher than the non-participants. Alternatively, rumination, social sharing and expressive suppression were not statistically significant between groups.

Considering the dataset comprised over 400 samples and the data for the reappraisal variable were likely normally distributed, an Independent Samples *t*-test was also performed to verify the consistency of the results previously observed in the Mann-Whitney U-test. The findings depicted a similar pattern, indicating that young adults who participated in sports had a significantly higher average score in reappraisal use (*M* = 4.22, SD = 1.18) compared to their counterparts who did not participate in sports (*M* = 3.82, SD = 1.28), despite a small Cohen’s d effect size (*p* = 0.001, *d* = 0.33).

Subsequently, ERQ data was examined using procedures similar to those applied to ERS data. First, the mean score of cognitive reappraisal (*M* = 4.75) was significantly higher than expressive suppression (*M* = 45), *t*(430) = −5.19, *p* < 0.001, 95% CI [−0.41, −0.18]. Second, Levene’s test results for cognitive reappraisal (*F* = 0.21, *p* = 0.645) and expressive suppression (*F* = 0.77, *p* = 0.378) did not show statistical significance, and the normality test did not reveal any severe deviations, thereby satisfying the assumption tests. The Independent Samples *t*-test was then conducted for ERQ to determine whether individuals engaged in sports showed a difference in the tendency to use cognitive reappraisal and expressive suppression strategies. The results revealed a significant difference in cognitive reappraisal, *t*(429) = −1.996, *p* = 0.047, *d* = −0.21, 95% CI [−0.41, −0.003], and non-significant in expressive suppression *t*(429) = −1.39, *p* = 0.166, *d* = −0.14, 95% CI [−0.40, 0.07]. The findings in ERQ indicated that young adults who took part in sports had a significantly higher mean score in cognitive reappraisal (*M* = 4.81, SD = 1.02) compared to those who did not participate in sports (*M* = 4.61, SD = 1.01), despite a small Cohen’s d effect size (*d* = 0.21).

The results from both the ERS and ERQ suggest that individuals who participate in sports are more likely to use reappraisal skills.

### 3.3 The role of competitive settings in sports play

This study aimed to assess the influence of competitive sports settings on the relationship between sports participation and reappraisal skills. To the extent that sports participation can take various forms, the analysis in this study categorized sports participation into two main groups: competitive (school or club team, competitive social) and non-competitive (non-competitive social and solo).

**FIGURE 1:**
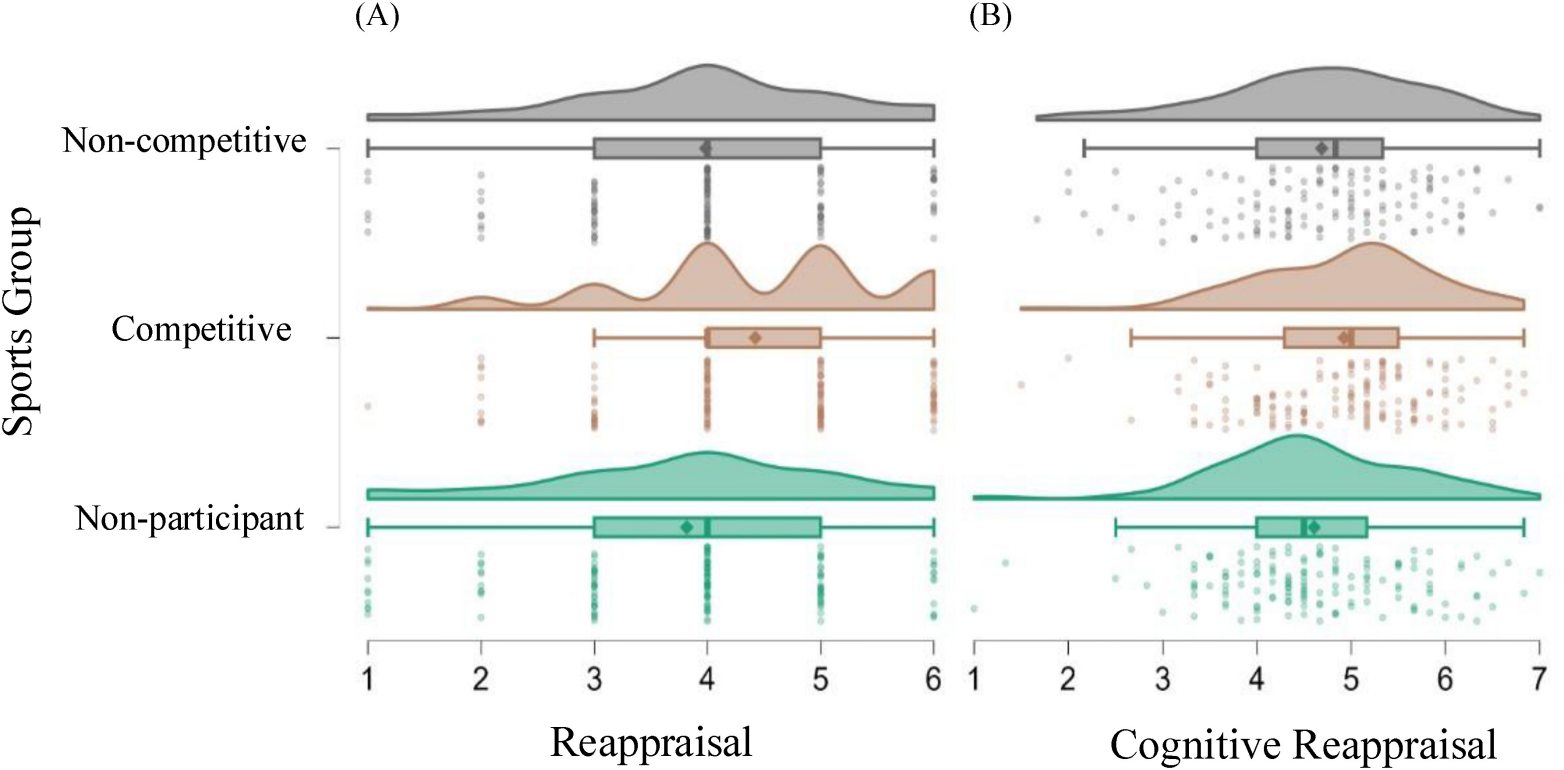
Means comparison of reappraisal (A) and cognitive reappraisal (B) by sports group.

A one-way ANOVA was conducted to compare the means of the three groups: non-participant (*n* = 142), competitive (*n* = 156), and non-competitive (*n* = 133). The variances of the ERS reappraisal of the three groups were compared. A Levene’s test of homogeneity of variances indicated that the variances were homogenous, *F* = 1.46, *p* = 0.232. The results of the means comparison showed a significant difference in reappraisal between groups, *F*(2, 428) = 10.21, *p* < 0.001, η^2^ = 0.05, indicating that at least one of the group means differed from the others. As shown in Figure 1A, Tukey’s HSD *Post-hoc* tests revealed significant pairwise differences in reappraisal between competitive and non-competitive groups, with an average difference of 0.44 (*p* = 0.006), and between competitive and non-participant groups, with an average difference of 0.61 (*p* < 0.001). According to the findings, the competitive group (*M* = 4.42, SD = 1.14) showed the highest tendency to employ reappraisal, followed by the non-competitive (*M* = 3.99, SD = 1.18) and the non-participant groups (*M* = 3.82, SD = 1.28). The medium effect size suggests that the significant mean differences of 0.44 and 0.61 reflect meaningful variations in the tendency to use reappraisal between competitive and other groups.

A separate one-way ANOVA was conducted for the ERQ, with cognitive reappraisal as the dependent variable. Figure 1B indicates a significant difference among the three groups in their use of cognitive reappraisal, with *F*(2, 428) = 3.97, *p* = 0.02, and η^2^ = 0.02. The results show a contrast only between the competitive group (*M* = 4.92, SD = 0.94) and non-participant (*M* = 4.61, SD = 1.01). Contrary to the findings in ERS, no statistically significant difference was observed in the use of cognitive reappraisal between the competitive and non-competitive groups. The results partially suggest that competitive sports may be associated with a greater tendency to use reappraisal skills, underscoring the impact of competitive environments on reappraisal strategies. Nevertheless, other potential variables, such as sports duration, may also be at play to influence the relationship.

### 3.4 The Relationship between sports duration and reappraisal use

Out of the 156 competitive sports players, 64 have participated in sports for over a year, while 62 out of 133 non-competitive sports players have played sports for a similar duration. An Independent Samples *t*-test was performed to analyze the relationship between the number of months playing sports and the attempt to use cognitive reappraisal. The assumption tests for normality and homogeneity of variance were confirmed (*F* = 0.36, *p* = 0.549). The analysis revealed no statistically significant difference in the use of cognitive reappraisal based on the number of months of sports participation. This finding suggests that the sports duration did not have a significant impact on the use of cognitive reappraisal among the sports participants.

### 3.5 Correlation between depression symptoms and cognitive reappraisal

Given that sports duration did not affect the use of cognitive reappraisal, we further explored whether other determinants in a competitive sport might impact the use of cognitive reappraisal (CR). A Multiple Linear Regression model was then used to assess the contributions of age, gender, number of negative life events (NLE), and experiencing depression symptoms (DS). The analysis aimed to determine the model’s overall fit and provide a comprehensive understanding of how these predictors collectively influence reappraisal skills.

Prior to assessing the model’s predictive capability, an examination of its foundational assumptions was conducted to ensure the integrity of the analysis. The residual plot, Q-Q plot, and histogram indicated no deviations from linear expectations, satisfied the normality criterion, and demonstrated no violation of homoscedasticity. Moreover, the Variance Inflation Factor (VIF) for each predictor was well below the threshold of 3, alleviating concerns regarding multicollinearity. Furthermore, the Durbin-Watson statistics of 2.01 effectively negated any autocorrelation among residuals, confirming the independence of errors.

The overall fit of the model was statistically significant. Analysis reveals that model H_1_ accounted for a significant portion of the variation in cognitive reappraisal, as evidenced by *F*(4, 395) = 2.71, *p* = 0.03. Model H_1_, which includes gender, total number of NLE, and DS, showed a considerable improvement over the null model. The results suggested that at least one predictor significantly impacts CR. In addition, model H_1_ explained approximately 2.7% of the variance in CR, R^2^ = 0.027, R^2^-change = 0.026, *F*-change (3, 395) = 3.52, *p* = 0.015. Notably, the analysis in Table 2 indicates that DS emerged as a significant predictor (*B* = −0.03, SE = 0.01, *p* = 0.002, 95% CI [−0.05, −0.01]). However, there was no significant association between age and CR (*B* = 0.02, SE = 0.02, *p* = 0.5, 95% CI [−0.03, 0.06]), nor between gender and CR (*B* = 0.03, SE = 0.10, *p* = 0.783, 95% CI [−0.17, 0.23]), as well as the total number of NLE and CR (*B* = 0.02, SE = 0.02, *p* = 0.521 95% CI [−0.03, 0.06]). The negative correlation between DS and CR was confirmed, with *r*(408) = −0.16, *p* = 0.001, according to Pearson’s correlation analysis.

**TABLE 2:** Coefficients of the multiple linear regression model.

Model	Variables	Unstandardized	Standard error	Standardized	t	Sig.	95% CI	
							Lower	Upper
H_0_	(Intercept)	4.465	0.543		8.219	<0.001	3.397	5.533
	Age	0.013	0.024	0.026	0.526	0.599	−0.035	0.061
H_1_	(Intercept)	4.656	0.56		8.314	<0.001	3.555	5.757
	Age	0.016	0.024	0.034	0.675	0.5	−0.031	0.064
	Gender	0.028	0.103	0.014	0.276	0.783	−0.174	0.231
	NLE	0.016	0.024	0.035	0.642	0.521	−0.032	0.064
DS	DS	−0.029	0.009	−0.17	−3.159	0.002	−0.046	−0.011

Null model includes Age. H_1_ model includes gender, the total number of negative life events, and endorsement of depression symptoms. The sample size was reduced to 400 after excluding 8 responses to other gender identities and 23 refused responses to depression screening questions.

The findings suggest that as young adults were more likely to experience DS, they tended to employ fewer CR strategies, while those who made more attempts to use CR had a lower likelihood of experiencing DS. Additionally, there was no statistically significant difference between the groups of sports non-participants, competitive sports participants, and non-competitive sports participants, as indicated by the results of the analysis of variance, *F*(2, 405) = 1.15, *p* = 0.318.

In light of the fact that young adults experiencing DS tend to use CR strategies less frequently, we are interested in investigating whether this relationship might impact the association between participating in competitive sports and using CR. To explore this potential relationship, this study employed an ANCOVA as an extension of a one-way ANOVA to reassess the variance in mean values among competitive, non-competitive, and non-participant groups while controlling for DS as a covariate. The covariate, DS, was significantly related to the CR, *F*(1, 404) = 11.57, *p* < 0.001, η^2^ = 0.03. When the covariate was added, the amount of variation accounted for competitive settings increased to 8.65 units and the unexplained variance reduced to 400.86. Moreover, there was a significant effect of competitive settings on CR after controlling for the effect of DS, *F*(2, 404) = 4.37, *p* = 0.013, η^2^ = 0.02. The effect size remained, approximately 2% of the variance in CR was influenced by competitive settings in sports. The results of the simple contrast indicated that competitive sports participants exhibited significantly higher CR values compared to non-participants in sports (contrast 1, *p* = 0.009). These differences were not observed between non-competitive sports participants and non-participants (contrast 2, *p* = 0.875).

**FIGURE 2:**
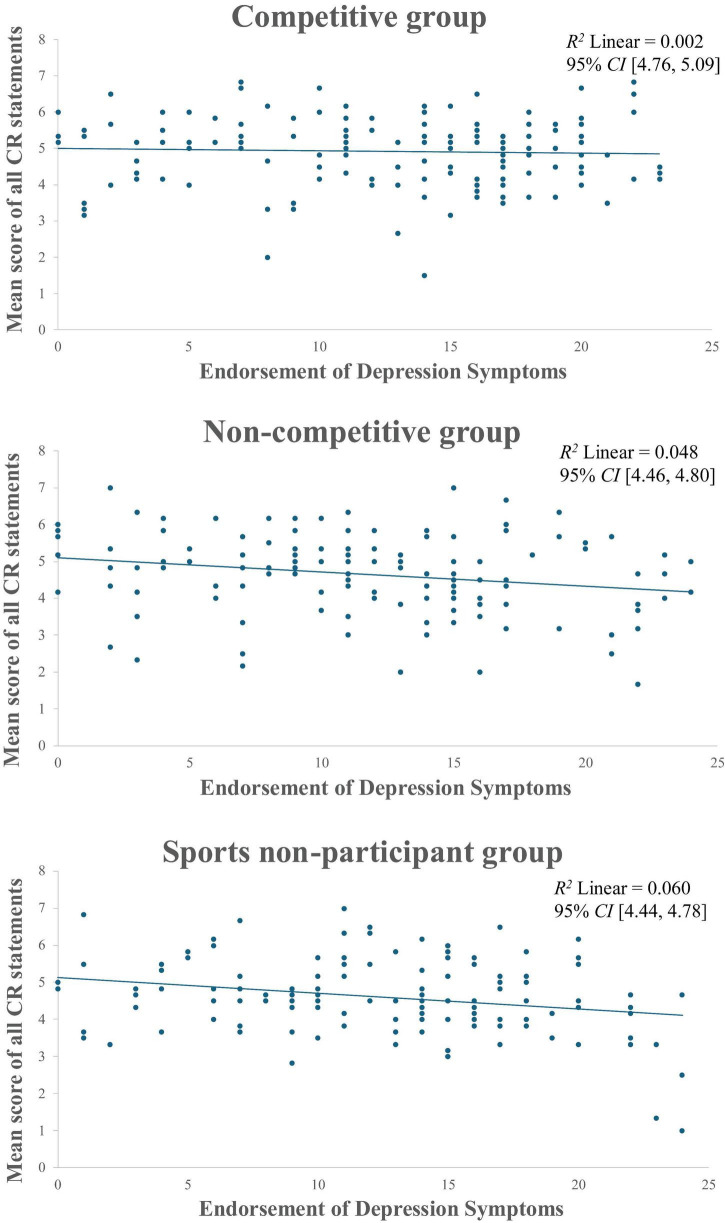
The correlation between CR mean and depression by sports groups.

Following the main ANOVA, a subsequent Tukey’s HSD test showed a significant difference not only between the competitive and sports non-participant groups (*p* = 0.024) but also between the competitive and non-competitive groups (*p* = 0.04). The difference was attributed to the reduced marginal mean in the non-competitive group (M_difference_ = −0.06) and a slight increase in the competitive group (M_difference_ = 0.01). In contrast, the marginal mean of the non-participant group remained unchanged. Furthermore, the correlation analysis between DS and CR within different sports groups is worth noting. As illustrated in Figure 2, an observation is that the negative correlation between DS and CR was relatively less pronounced in the competitive group (R^2^ Linear = 0.002) compared to the other two groups (non-competitive: R^2^ Linear = 0.05; non-participant: R^2^ Linear = 0.06). These findings explain only 0.2% of the variation in reappraisal is accounted for by DS, compared to 6% in non-participant and 5% in non-competitive groups. Taken together, it is essential to consider the significance of competitive environments in sports participation when examining the use of reappraisal skills.

### 3.6 Gender differences in depression symptoms, sports participation and emotion regulation strategies

In the context of DS potentially influencing CR, a separate Independent Samples *t*-test was undertaken to investigate whether there was a gender difference in DS. The results showed that females (*M* = 12.75, SD = 5.77) reported significantly higher DS scores than males (*M* = 11.56, SD = 6.30), with *t*(398) = 1.97, *p* = 0.05, *d* = 0.2, 95% CI [0.002, 2.38]. In addition, to examine the presence of gender differences in sports participation, this study conducted a Chi-square test to explore the relationship between gender and sports participation. The results in Table 3 demonstrate a significant correlation: Females showed lower engagement in competitive sports, χ^2^(2) = 17.7, *p* < 0.001, Cramer’s *V* = 0.21, suggesting a moderate strength of association. The findings suggest that females are more likely to experience DS and participate less in competitive sports than males. In relation to emotion regulation strategies, females reported a significantly greater tendency to use rumination compared to males, *t* = 1.97, *p* = 0.049, 95% CI [0.001, 0.50], whereas males demonstrated a significantly greater tendency to use reflection than females, *t* = −2.18, *p* = 0.03, 95% CI [−0.51, −0.03]. No significant differences were observed in other emotion regulation strategies.

**TABLE 3:** Frequencies for sports group by gender identity.

Gender	Sports group	Frequency	Percent	Cumulative percent
Female	Competitive	63	27.6%	27.6%
	Non-competitive	82	36.0%	63.6%
	Non-participant	83	36.4%	100.0%
Male	Competitive	92	47.2%	47.2%
	Non-competitive	47	24.1%	71.3%
	Non-participant	56	28.7%	100.0%
Others	Competitive	1	12.5%	12.5%
	Non-competitive	4	50.0%	62.5%
	Non-participant	3	37.5%	100.0%

Others include transgender females, transgender males, individuals with other gender identities, and those who prefer not to specify.

## 4 Discussion

### 4.1 Sports participation and reappraisal use

To understand how competitive sports impact the relationship between sports participation and reappraisal use, we first investigated whether sports participation would be associated with reappraisal among young adults. The data supported the hypothesis that young adults who participated in sports would have a greater tendency to use reappraisal than those who did not, with a mean difference of 0.2–0.4 in ERQ and ERS tests, respectively. These findings are consistent with previous research demonstrating a positive link between fitness dancing and cognitive reappraisal. For instance, it was shown that exercises could enhance cognitive reappraisal skills among Chinese female postgraduates at Northeastern University (Liu et al., 2022). These results can be explained by the fact that sports often involve challenging situations and performance pressure, which may demand effective emotion regulation. Similarly, individuals continuously participating in sports may learn and use reappraisal techniques to enhance their performance (Balk et al., 2013).

### 4.2 Competitive vs. non-competitive sports

We examined the association between sports participation and reappraisal, particularly in a competitive environment. Our second hypothesis posited that individuals engaged in competitive sports would attempt to use more reappraisal skills than those in non-competitive sports or non-participants. The findings from the ERS test supported this hypothesis by demonstrating that competitive sports participants displayed a higher tendency to use reappraisal. However, the data from the ERQ test generally upheld the hypothesis, albeit with one exception: young adults in competitive sports showed a higher tendency to employ reappraisal only compared to non-participants, not the non-competitive groups. These findings suggest that various factors, such as depression experience or constant negative life events that trigger emotions, may also factor into reappraisal use. There are a couple of plausible pathways to consider.

One plausible pathway in exploring the relationship between sports participation and reappraisal use is that competitive sports provide a structured environment where individuals can practice and refine reappraisal skills. Due to the high-pressure situations inherent in competitive sports, individuals may also be required to frequently reappraise challenging scenarios to maintain focus and build resilience from recovering from mistakes to improve performance (Balk et al., 2013; Tamminen et al., 2016). Learning from failure may be associated with working memory, which helps enhance the ability to use reappraisal strategies (Zhao et al., 2023) beyond the sports context to daily life. From a psychological perspective, competitive sports may serve as a practical training ground for developing reappraisal skills and result in reframing day-to-day adverse events to reduce negative emotions.

In the alternative pathway, individuals with solid reappraisal skills may naturally be drawn to competitive sports. It is plausible that those with better reappraisal skills have less fear of failure. That said, they are more enthusiastic about the competitive nature of sports. On the other hand, individuals with weaker reappraisal skills may have less desire to engage in competitive sports. They may feel shame and lack the confidence and motivation to face more challenges after experiencing failure (McGregor and Elliot, 2005). From a psychological perspective, the demanding nature of competitive environments may naturally attract individuals skilled in managing their emotions and using reappraisal as a coping strategy. They may find competition fulfilling and motivating for better performance.

Beyond these two pathways, it is important to consider the influence of third variables that may shape the observed relationship between sports participation and reappraisal use. A third variable, such as socioeconomic status (SES), social networks, physical health and sleep quality (Glavin et al., 2022), could influence the relationship between sports participation and reappraisal use. For instance, individuals with strong financial stability and supportive networks may find it easier to access competitive sports opportunities. Emotional encouragement from teammates can also boost their mood (Sahli et al., 2020) and help them interpret challenging situations positively. Moreover, individuals with better physical health are less prone to injuries, which allows them to sustain sports participation. Similarly, those with sufficient sleep tend to experience better mood and energy, making them more likely to engage in sports actively. From a psychological perspective, a healthy body promotes a clear mind and stable mood, which can positively impact sports participation and the tendency to use reappraisal as a coping mechanism.

### 4.3 Duration of sports participation

Despite the expected connection between reappraisal and competitive sports environments, the Independent Samples *t*-test results showed that the duration of playing sports did not significantly affect the relationship between reappraisal and sports participation. Therefore, the data did not support the third hypothesis, suggesting that the association between sports participation and reappraisal use would become more robust with longer-term sports participation. Additionally, the results of the present study’s multiple regression linear analysis indicated no significant correlation between NLE experienced by young adults and reappraisal. These findings aligned with the study by Roos and Bennett (2023), which highlights the interaction between stressful life events, depressive symptoms, and the role of reappraisal in young adults. The authors observed a positive association between stressful life events and depressive symptoms in young adults, but found no direct association with attempts to reappraise and efficacy. For example, young adults reported lower levels of depression symptoms when exposed to fewer stressful life events and higher levels when exposed to more stressful events.

### 4.4 Depression symptoms and gender

Depression symptoms (DS) in early adulthood emerged as a significant predictor in our study, explaining a substantial portion of the variance in reappraisal. The result underscores a significant inverse association between reappraisal use in young adults and their likelihood of experiencing DS. In other words, young adults who experienced DS tended to engage in less reappraisal, whereas those who attempted more to reappraise their daily challenges experienced fewer depression symptoms. It was consistent with the previous literature’s negative correlation between perceived stress and cognitive reappraisal (Wessa et al., 2024; Xu et al., 2020).

After taking DS into account, it was observed that young adults participating in competitive sports used significantly more reappraisal than non-competitive sports participants, in addition to the non-participants. In the new relationship between competitive and non-competitive groups, it is presumed that the competitive group may be more responsive to stressful situations, leading to a heightened sensitivity to reappraisal use. Contrary to the presumption, the results in the negative correlation between reappraisal and depression symptoms highlight the differential impact of depression on the use of reappraisal across different sports groups. While depression may have a minimal direct impact on reappraisal among young adults engaged in competitive sports, it appears to be more influential in the non-participant and non-competitive sports groups.

Furthermore, when DS was controlled at the same mean DS value of 12.30, the estimated marginal mean of the reappraisal use showed a slight increase for both competitive (M_difference_ = 0.002) and non-participant groups (M_difference_ = 0.004), while it decreased significantly for the non-competitive group (M_difference_ = −0.06). Considering the data, this reduction in reappraisal use in the non-competitive group may be explained by an interaction between DS and other factors, such as socioeconomic or severity of DS, influencing reappraisal use.

Finally, in line with various research, this study has shown no significant differences between genders in their use of reappraisal (Gross and John, 2003; Perchtold et al., 2019). However, it is noteworthy that females tend to be prone to feeling depressed and participate less in competitive sports than their male counterparts. Considering the distress and physical inactivity that would hinder the attempt of reappraisal, it was theorized that females would use the reappraisal strategy less frequently than young men. The results indeed contradict this hypothesis. One potential explanation could be that females are more flexible in using emotion regulation strategies, including adaptive and maladaptive (Goubet and Chrysikou, 2019). Despite feeling more depressed and lower participation in competitive sports, females may possess other emotion regulation skills that could simultaneously enhance the frequency of reappraisal use. In line with a study by Lask et al. (2021), females in the current study were more likely to focus on repetitive negative thinking than men. However, they may be more likely to seek social support, express their emotions, and discuss personal problems with their same-sex friends in response to stressors (Nielson et al., 2020; Taylor et al., 2000). The emotional and informational support from friends and family may potentially help reframe challenging situations from different perspectives.

## 5 Conclusion

This study indicated that participating in sports was associated with a greater tendency to use reappraisal among young adults. Additionally, this study further investigated the role of competitive environments in the context of sports and the self-perceived frequency of reappraisal use. Although the effect size was relatively small, analyzing the relationship between participating in competitive sports and the tendency to use reappraisal yielded statistically significant results. Notably, this study also found that depression symptoms, as opposed to gender and sports duration, play a pivotal role in the potential relationship between sports participation and the tendency to use reappraisal.

While our study did not reveal statistically significant differences in the experience of negative life events (NLE) and depression symptoms between sports participants and non-participants, we observed that sports participants reported a marginally higher incidence of NLE in the past 3 months, such as the loss of a close family member or major financial strain, compared to non-participants. Despite this, sports participants, on average, reported a lower likelihood of experiencing DS, underscoring the positive impact of sports participation on mental health.

Regarding emotion regulation strategies, our study found that young adults predominantly rely on distraction and reflection as their primary coping tactics to deal with their emotions. In contrast, reappraisal and social sharing were the strategies that were utilized the least. Several potential explanations exist for the decreased use of reappraisal and social sharing strategies. First, excessive internet access may drive individuals to seek instant stress relief due to difficulties in regulating emotions (Gioia et al., 2021). Second, young adults may prefer immediate relief strategies and opt for distraction from distressing emotions rather than using reappraisal, which may demand effortful attempts (Milyavsky et al., 2019). Third, as young adults transition to adulthood, changes in social support networks may further exacerbate the prevalence of reappraisal (Metts and Craske, 2023).

## 6 Limitations and future directions

In this study, we are limited to observing the strength of the association between the variables. It remains inconclusive whether sports participation induces changes in the tendency to use reappraisal or whether variations in reappraisal influence interest in sports. Additionally, a third variable may underpin both phenomena, requiring further investigation to clarify these relationships. Several limitations of the current study should be considered when interpreting the findings. These include the correlational design, reliance on self-report, and ambiguity in sports categorization.

Notwithstanding these limitations, the current study contributes significantly to the understanding of emotion regulation among young adults by addressing substantial gaps in knowledge regarding the relationship between reappraisal use and sports participation, particularly within competitive environments. Moreover, longitudinal and experimental research examining the relationships between reappraisal, sports participation, and exposure to stressful life events over time would be valuable for elucidating directionality and establishing causal sequences among these variables. Furthermore, the potential influence of cultural differences on reappraisal was not assessed in this study. Recruiting participants from diverse backgrounds, such as socioeconomic status, gender, and culture, in future research to explore broader generalizability.

## Data Availability

The raw data supporting the conclusions of this article will be made available by the authors, without undue reservation.
